# A graph convolutional network for predicting COVID-19 dynamics in 190 regions/countries

**DOI:** 10.3389/fpubh.2022.911336

**Published:** 2022-08-03

**Authors:** Sumiko Anno, Tsubasa Hirakawa, Satoru Sugita, Shinya Yasumoto

**Affiliations:** ^1^Graduate School of Global Environmental Studies, Sophia University, Tokyo, Japan; ^2^Chubu Institute for Advanced Studies, Chubu University, Kasugai, Japan

**Keywords:** COVID-19, deep learning, graph convolutional network, predicting, public transportation

## Abstract

**Introduction:**

Coronavirus disease (COVID-19) rapidly spread from Wuhan, China to other parts of China and other regions/countries around the world, resulting in a pandemic due to large populations moving through the massive transport hubs connecting all regions of China *via* railways and a major international airport. COVID-19 will remain a threat until safe and effective vaccines and antiviral drugs have been developed, distributed, and administered on a global scale. Thus, there is urgent need to establish effective implementation of preemptive non-pharmaceutical interventions for appropriate prevention and control strategies, and predicting future COVID-19 cases is required to monitor and control the issue.

**Methods:**

This study attempts to utilize a three-layer graph convolutional network (GCN) model to predict future COVID-19 cases in 190 regions and countries using COVID-19 case data, commercial flight route data, and digital maps of public transportation in terms of transnational human mobility. We compared the performance of the proposed GCN model to a multilayer perceptron (MLP) model on a dataset of COVID-19 cases (excluding the graph representation). The prediction performance of the models was evaluated using the mean squared error.

**Results:**

Our results demonstrate that the proposed GCN model can achieve better graph utilization and performance compared to the baseline in terms of both prediction accuracy and stability.

**Discussion:**

The proposed GCN model is a useful means to predict COVID-19 cases at regional and national levels. Such predictions can be used to facilitate public health solutions in public health responses to the COVID-19 pandemic using deep learning and data pooling. In addition, the proposed GCN model may help public health policymakers in decision making in terms of epidemic prevention and control strategies.

## Introduction

Most early cases of the novel coronavirus disease-2019 (COVID-19) have been linked to exposure to wildlife at the Huanan Seafood Wholesale Market in Wuhan, China (1). However, an exponential increase in the number of non-linked cases was identified in late December 2019 (2). Given the global spread of COVID-19, public transport systems that facilitate transnational human mobility, e.g., air travel, railroads, and automobiles, should be considered potential risk factors in the COVID-19 pandemic context.

According to current evidence, many early patients worked in or visited the market, where bats, snakes, and mink are sold. These animals are considered to be natural or intermediate hosts of severe acute respiratory syndrome coronavirus 2 (SARS-CoV-2), and the sale of such animals suggests that transmission of the virus to humans began during the first phase of the epidemic (3). In addition, the occurrence of human-to-human COVID-19 transmission occurred in clusters of family members, including relatives and friends with intimate contact with patients or incubation carriers and medical staff in hospitals (4, 5) *via* respiratory droplets or direct contact (2, 6). Furthermore, the epidemic spread rapidly in and outside China, there resulting in pandemic status due to large floating populations, including more than five million migrants residing in Wuhan who returned to their hometowns in other areas of China around the Chinese lunar New Year through massive transport hubs connecting all regions of China *via* rail (6, 7) and a major international airport (8). Despite closing the Huanan Seafood Wholesale Market on January 1, 2020, a lockdown of Wuhan on January 23, 2020, and subsequent travel restriction and border control (9), COVID-19 spread rapidly to other parts of China and other countries around the world (10).

The COVID-19 pandemic has had great impact on human life, society in general, and the world economy. COVID-19 will remain a threat until safe and effective vaccines and antiviral drugs have been developed, distributed, and administered around the world. There is an urgent need to establish effective implementation of non-pharmaceutical interventions for appropriate prevention and control strategies. We believe that predicting future COVID-19 cases can help detect infections and disrupt the COVID-19 (11) chain of transmission by supporting decisive action in our responses to future pandemics (12).

Various models have been developed to predict future COVID-19 cases using mathematical approaches (13), machine learning (14), and deep learning (15). Moein et al. applied the susceptible-infected-recovered model to predict the outbreak of COVID-19 and discovered that the model was unable to forecast the actual spread and pattern of the epidemic in the long term. They suggested that more sophisticated modeling approaches in line with more precise epidemiological and biomedical data are urgently required to make the pandemic forecasting feasible (13). Rath et al. used multiple linear regression models to forecast the forthcoming days of active cases of COVID-19 in Odisha and India using daily positive, recovered, and deceased cases. Although it was found to be an effective way to forecast the cases, limitations of the model were discussed, including the collection of more independent variables and information and ways to find the number of contact tracing cases (14). Xu et al. used three deep learning models, namely, convolutional neural network, long short-term memory, and convolutional neural network-long short-term memory with COVID-19 data of the following three highly impacted countries: Brazil, India, and Russia to predict the number of COVID-19 cases. They found that the long short-term memory model had the highest performance in forecasting accuracy compared with other models. Since the model prediction was high on datasets for the three countries, they suggested the need for a larger quantity of training data to achieve more accurate results and support the global fight against the pandemic (15).

These models struggle to deal with the epidemiological process of the disease that spreads across countries or continents and is spatially heterogeneous. A COVID-19 prediction model must consider an important characteristic of the pandemic. COVID-19 is transmitted from person to person along with human mobility *via* public transportation (6–8) and in local environment. It is important to model contagion dynamics on complex networks (16). To address this issue, we proposed a graph convolutional network (GCN) model that captures latent geographical flow of people *via* a public transportation network represented as a graph comprising nodes and edges.

## Materials and methods

Our experimental process involved collecting and preprocessing data, conducting experiments, and assessing the performance of the proposed GCN model.

### Data collection

Data on daily new confirmed COVID-19 cases were collected from Our World in Data (17). In addition, data on the public transportation networks were obtained from the OAG flight data (18) and Natural Earth (19), which capture latent transnational human mobility. The OAG flight data include airplane operation records, i.e., takeoff and landing airports. Here, the data period was November 2019, just before the COVD-19 pandemic. In addition, the geographical boundary data, i.e., region and country, were obtained from Natural Earth (19).

### Data preprocessing

The collected data were preprocessed to create a dataset to be used in our deep learning experiments. We calculated the latitude and longitude of the capital city of each region or country from a digital map, created an ISO region/country code list, and created a data that holds two values, i.e., the latitude and longitude of the capital city in each region or country and the ISO region/country code, as a pair. This data is made to correspond to the data on COVID-19. In this study, we selected 190 regions and countries with the highest number of infected people. Note that the number of daily new confirmed COVID-19 cases was normalized from 0 to 1.

We constructed three types of graphs with nodes and identified pairs of nodes (edges including self-loops) using the collected data and assigned attributes to each node and edge. The nodes in the graphs represent regions and countries that contain daily new confirmed COVID-19 cases, and the edges represent the airways (Supplementary Figure S1A), railways (Supplementary Figure S1B), and roads that connect regions and countries (Supplementary Figure S1C). Note that we assumed two regions and countries that are adjacent to each other are connected by roads.

### Graph convolutional networks

The preprocessed datasets were used to train the proposed GCN model, which operates on graphs and aggregates their structural information (20). The goal of a GCN model is to learn a function of features in a graph *G* = (*V, E*) that takes the following as input (i) a feature *x*_*i*_ for each node *i* summarized in an *N* × *D* feature matrix *X* (where *N* is the number of nodes, and *D* is the number of input features), and (ii) a representative description of the graph structure in matrix form (typically in the form of an adjacency matrix *A* or some function thereof). The GCN model produces a node-level output *Z* (i.e., an *N* × *F* feature matrix, where *F* is the number of output features per node).

Thus, each neural network layer can be written as a non-linear function:


$$H^{\left(l+1\right)}\;=\;f\left(H^{\left(l\right)},A\right),\;$$

where *H*^(0)^ = *X* and *H*^(l)^ = *Z* (or *z* for graph-level outputs), and *l* is the number of layers. Note that specific models differ only in terms of how *f* (·,·) is selected and parameterized.

To sum all feature vectors of all neighboring nodes with self-loops and take the weighted average of all neighboring node features with self-loops, we employ a multilayer GCN with the following layer-wise propagation rule:


$$f\left(H^{\left(l\right)},A\right)\;=\;\sigma \left({\hat{D}}^{-1/2}\hat{A}{\hat{D}}^{-1/2}H^{\left(l\right)}W^{\left(l\right)}\right),\;$$

where *W*^(l)^ is a weight matrix for the *l*-th neural network layer. Here, *Â* = *A* + *I*, where *I* is the identity matrix, $\hat{D}$ is the diagonal node degree matrix of *Â*, and σ(·) is a non-linear activation function, e.g., ReLU.

Note that adding the identity matrix to adjacency matrix *A* can contribute to summing all of the feature vectors of all neighboring nodes along with self-loops. Normalizing adjacency matrix *A* by multiplying it with the inverse degree matrix *D* corresponds to taking the average of the neighboring node features (20, 21).

### Experiments

The experimental datasets consist of two parts. One part is the COVID-19 case data, which describe changes in the number of daily new confirmed COVID-19 cases from January 22, 2020 to September 17, 2021 in each region or country. We aggregated these data as a feature matrix, where each row represents a single region or country, and each column is the number of new confirmed COVID-19 cases in different time periods. Here, we aggregated the number of new confirmed COVID-19 cases in each region or country every 7 days. Another one is a 190 × 190 adjacency matrix that describes the spatial relationship between regions and countries. Here, each row represents a single region or country, and the values in the matrix represent the connectivity of airways, railways, or roads between regions and countries. This adjacency matrix only contains elements of 0 and 1, where 0 represents no link between regions/countries, and 1 represents the existence of a link.

**Figure 1:**
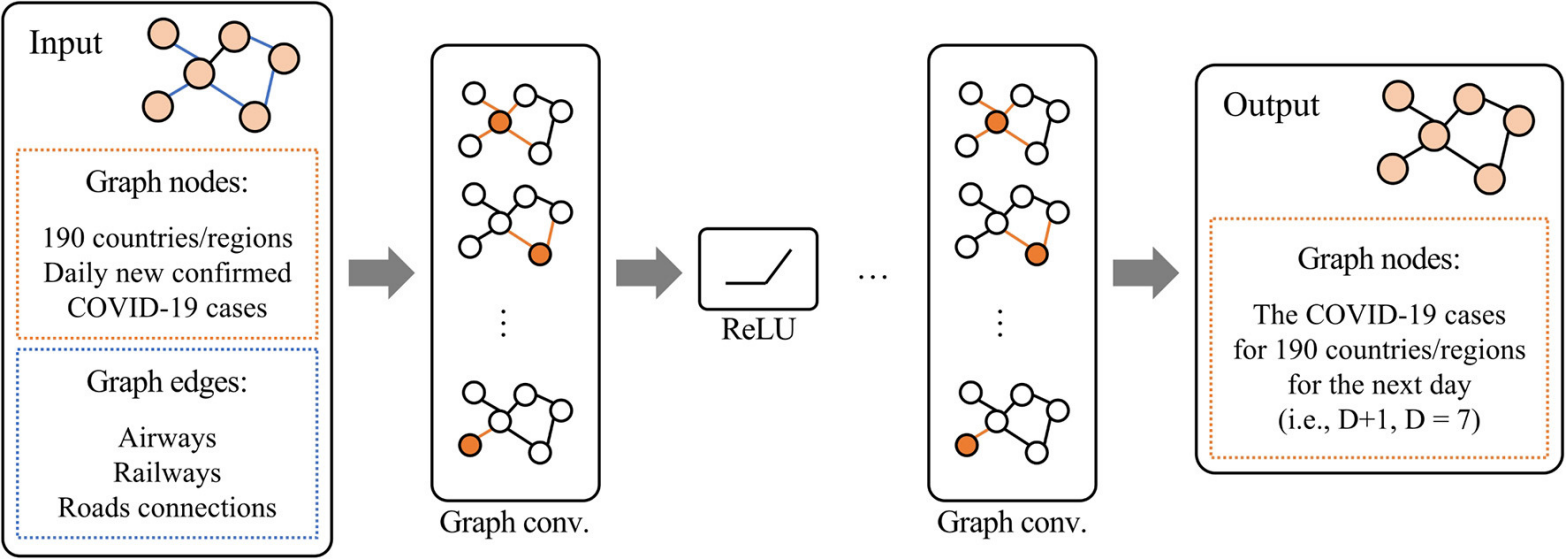
Proposed three-layer graph convolutional network model.

To predict the number of future COVID-19 cases on the eighth day (D + 1) based on data from the previous 7 days (D = 7) corresponding to 190 regions or countries, we trained the proposed three-layer GCN model (Figure 1) on the following graph data with the node features: (I) airways, (II) railways, (III) roads, (IV) airways, railways, and roads, (V) airways and railways, (VI) airways and roads, and (VII) railways and roads. Here, all activation functions were ReLU (except for the last linear layer). The dataset was split into 60% for training and 40% to evaluate the performance of the model. The model was trained for 500 epochs with 256 batches per epoch. In this experiment, the Adam optimizer was used with a learning rate of 0.01. The training model was applied to the test dataset to evaluate the performance of the model.

We compared the performance of the proposed three-layer GCN model to a multilayer perceptron (MLP) model excluding the graph representation on the COVID-19 case dataset. The prediction performance of the models was evaluated in terms of the mean squared error (MSE), the root mean squared error (RMSE), mean absolute error (MAE), root mean squared percentage error (RMSPE), and mean absolute percentage error (MAPE) for both the normalized test data and test data on the actual number of infected people. The MSE is defined as the mean or average of the square of the difference between the actual and predicted values. The RMSE takes square root for an MSE value. The MAE is the mean of the absolute values of the difference between the actual and predicted values. Note that the MSE, RMSE, and MAE take positive values, where a value close to zero implies that the corresponding prediction model obtains higher accuracy. The RMSPE is the mean of the percentages of squared error between actual and predicted values. It is an evaluation of the percentage by which the predicted value deviates from the actual value. Similar to RMSPE, the MAPE is the mean of the percentages of absolute error between actual and predicted values divided by the actual value. Note that the RMSPE and MAPE compute division by actual value. Therefore, we omit the computation of these metrics in case that the actual value is zero to avoid zero-division.

Note that experimental results may not be consistent due to the influence of the initial values of the network parameters. Thus, we conducted ten comparative experiments for the proposed GCN with each combination of (I) through (VII) and for the MLP, and we calculated the mean and standard deviation.

## Results

Table 1 shows the MSE, RMSE, MAE, RMSPE, and MAPE results for the GCN and the baseline MLP obtained on the normalized test data. Note that Table 1 lists the MSE, RMSE, and MAE for normalized values between 0 and 1. In terms of the MSE, RMSE, and MAE for the normalized values, the proposed GCN model achieved good performance compared to the MLP for all combinations of (I) through (VII). Focusing on the used graph connection in the GCN model, the results with airway demonstrates that the lowest prediction errors. In contrast, the results with all connections (i.e., airway, railway, and road) achieved higher prediction error than those of the other graph connections. These results indicate that connecting nodes as many as possible degenerates the prediction accuracy, and that selecting meaningful connections is important. Meanwhile, in terms of RMSPE and MAPE, the GCN with railway achieved better prediction performance. Here, the RMSPE and MAPE divide the difference between actual and predicted values by the actual value. If we compute these values for small actual values, these errors easily become high. We deal with the daily new confirmed COVID-19 cases. Depending on the day and region/country, actual values can be zero or extremely small. Hence, the GCN with railway connection accurately predicts the small number of COVID-19 cases.

**Table 1:** Mean squared error for normalized predictive values.

**Model**	**Graph connection**			**Evaluation metrics**									
	**Airway**	**Rail**	**Road**	**MSE**		**RMSE**		**MAE**		**RMSPE (%)**		**MAPE (%)**	
MLP				2.09E-03	±4.13E-03	3.58E-02	±2.85E-02	1.36E-02	±1.13E-02	1,772.48	±2,588.61	222.89	±288.79
GCN	✓			**6.73E-05**	±**5.42E-06**	**8.19E-03**	±**3.32E-04**	**2.21E-03**	±**2.20E-04**	76.03	±20.58	15.84	±4.85
		✓		8.28E-05	±7.87E-06	9.09E-03	±4.19E-04	2.41E-03	±3.54E-04	**73.00**	±**15.40**	**10.84**	±**4.23**
			✓	7.79E-05	±1.39E-05	8.79E-03	±7.67E-04	2.62E-03	±4.76E-04	110.27	±32.56	20.88	±8.39
	✓	✓		9.09E-05	±1.29E-05	9.51E-03	±6.58E-04	3.51E-03	±3.54E-04	134.17	±22.52	29.51	±6.21
	✓		✓	1.23E-04	±4.19E-05	1.09E-02	±1.80E-03	3.48E-03	±3.02E-04	169.56	±30.53	35.06	±6.01
		✓	✓	9.87E-05	±1.07E-05	9.92E-03	±5.37E-04	3.12E-03	±1.32E-04	142.89	±32.04	21.11	±3.13
	✓	✓	✓	1.84E-04	±7.45E-05	1.34E-02	±2.39E-03	5.15E-03	±1.23E-03	237.72	±67.29	49.91	±14.95

*Bold values denote the results of airway*.

For simplicity, we show compare the MSE, RMSE, and MAE results for the predicted and actual numbers of infected people in Table 2. Here, we computed these errors of infected people by inversely converting the normalized values. Note that the maximum number of daily new confirmed COVID-19 cases is 414,188. As for these errors converted to actual number of infected people, differences were depending on the type and combination of adjacency matrixes. The proposed GCN models predicted the number of infected people with an average MSE value from 27 to 76 (Table 2), and the MLP model predicted the number of infected people with an average MSE value of 865 (Table 2). The RMSEs of GCNs are between 3,000 and 5,500 and that of MLP is about 14,000. The MAEs of GCNs are between 900 and 2,000 and that of MLP is about 5,600. Thus, the proposed GCN model exhibited better graph utilization and better performance compared to the baseline MLP model in terms of both prediction accuracy and stability. In particular, the proposed GCN model with the adjacency matrix for airway demonstrated the best prediction accuracy.

**Table 2:** Mean squared error for the difference between predicted and actual numbers of infected people.

**Model**	**Graph connection**			**Evaluation metrics**					
	**Airway**	**Rail**	**Road**	**MSE**		**RMSE**		**MAE**	
MLP				865.52	±1,711.86	14,819.08	±11,784.90	5,633.10	±4,668.20
GCN	✓			**27.86**	±**2.24**	**3,393.88**	±**137.64**	**914.28**	±**91.33**
		✓		34.28	±3.26	3,763.86	±173.74	1,000.07	±146.66
			✓	32.25	±5.77	3,641.18	±317.85	1,085.26	±197.08
	✓	✓		37.63	±5.33	3,938.73	±272.45	1,453.42	±146.58
	✓		✓	50.75	±17.37	4,523.61	±746.62	1,442.33	±125.21
		✓	✓	40.90	±4.45	4,109.81	±222.28	1,292.86	±54.58
	✓	✓	✓	76.36	±30.84	5,535.77	±990.79	2,132.54	±509.85

*Bold values denote the results of airway*.

**Figure 2:**
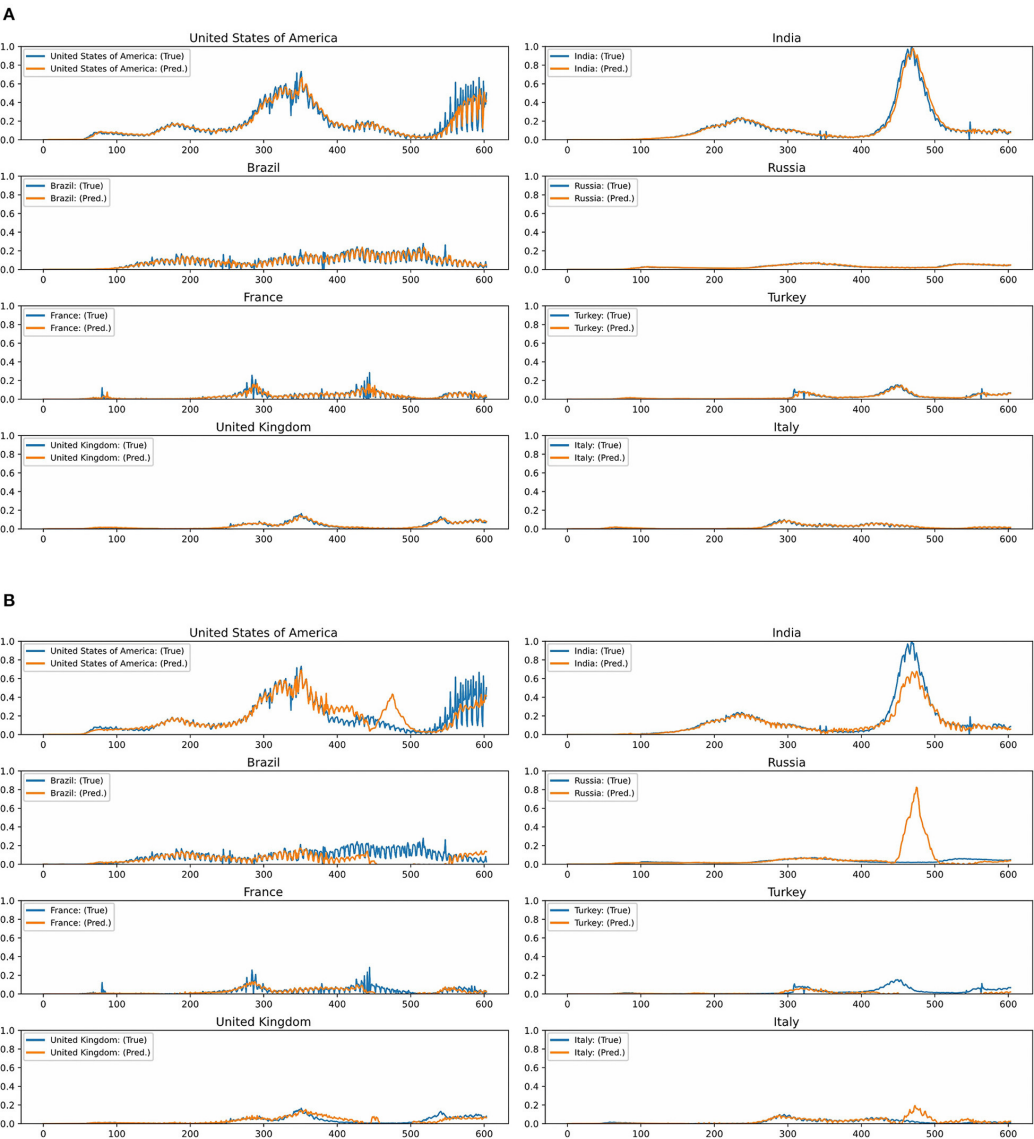
**(A)** Test predictions and ground truth values for the graph convolutional network model with the airway adjacency matrix in the top eight countries with the highest number of new infections. **(B)** Test predictions and ground truth values for the multilayer perceptron model in the top eight countries with the highest number of new infections.

Figures 2A,B show the predictions and ground truth values of the proposed GCN model with the airway adjacency matrix and the MLP model for the top eight regions/countries in terms of the cumulative number of infected cases and deaths as of January 20, 2022. Here, the orange lines represent the prediction results obtained by the model on the normalized test data, and the blue lines represent the ground truth values (i.e., the normalized COVID-19 case data). As can be seen, in this case, the proposed GCN model exhibits the smallest generalization gap between the predictions and ground truth. Yet, in the countries with rapidly changing number of cases, the model may face challenges in capturing the changing trends. In addition, the curves in the predictions and ground truth are similar, which indicates higher performance in regions/countries with low fluctuation in the number of COVID-19 cases, e.g., the United Kingdom, Russia, and Italy (Figure 2A). For the MLP model, deviations between the model's predictions and the ground truth are observed in seven countries (i.e., United States of America, India, Brazil, Russia, France, Turkey, and United Kingdom), with the exception of Italy (Figure 2B).

Supplementary Figures S2A,B show the learning curves for the loss function of the proposed GCN model with the airway adjacency matrix and the MLP model, respectively. For the proposed GCN model, after 50 epochs, the loss curves of the training and test sets tend to converge with no sign of overfitting. With the MLP, the loss curves for the test data increase rapidly early during training. Supplementary Figure S2A shows that the proposed GCN model obtains better stability and a smaller generalization gap between the training and test loss than the MLP.

## Discussion

Our findings suggest that the proposed GCN model is useful in terms of predicting COVID-19 cases at both regional and country levels in terms of MSE, RMSE, MAE, RMSPE, and MAPE. We found that the proposed GCN model outperformed the MLP model, and the proposed GCN model was trained in a stable manner. We believe that the proposed GCN model outperformed the MLP model because it better exploits the graph structural information about the public transportation network by effectively extracting meaningful features from the sequential graph data *via* multiple spatiotemporal graph convolution units. The proposed GCN model may offer positive contribution to the prediction of the future COVID-19 cases and the detection of potential factors influencing the COVID-19 pandemic. The experimental results demonstrate that the proposed GCN models with the adjacency matrix for public transportation networks improve prediction accuracy compared with the MLP model. These results suggest that human mobility *via* public transportation may continue to introduce infection to other regions/countries. Given relatively recent epidemics, e.g., severe SARS, public transportation (particularly commercial air travel) is considered a potential risk factor in the rapid global spread of infectious diseases (22). Wuhan serves massive transport hubs including not only airways but also roads and railways, passing through the cities and connecting to other major regions/countries. The transport hubs may have accelerated the COVID-19 pandemic.

We expect that graph-based deep learning with data pooling will provide digital health solutions to public health responses to the COVID-19 pandemic. The proposed GCN model will help in predicting COVID-19 dynamics (that may be caused by variants of concern) at regional and national levels. Implementing border control measures in regions and countries having the variants and testing for COVID-19 infection (23) will help to control the spread of any variant of COVID-19 infection. In addition, integrating deep learning techniques into early warning systems may help realize effective alert systems and the generation of maps identifying locations with high risk of infection, which can be used to guide appropriate responses to emerging and reemerging infectious diseases with pandemic potential.

## Data availability statement

The original contributions presented in the study are included in the article/Supplementary material, further inquiries can be directed to the corresponding author.

## Author contributions

SA designed the study and drafted the original manuscript. SA and TH conceptualized the analysis and conducted the experiment in deep learning and did interpretation. SS and SY collected the data and processed for the experiment. All authors reviewed, discussed, and approved the final manuscript for submission.

## Funding

This work was supported by the Collaboration Research Program of IDEAS, Chubu University IDEAS202109.

## Conflict of interest

The authors declare that the research was conducted in the absence of any commercial or financial relationships that could be construed as a potential conflict of interest.

## Publisher's note

All claims expressed in this article are solely those of the authors and do not necessarily represent those of their affiliated organizations, or those of the publisher, the editors and the reviewers. Any product that may be evaluated in this article, or claim that may be made by its manufacturer, is not guaranteed or endorsed by the publisher.

## Supplementary material

Supplementary Figure S1 Public transport network as a graph: **(A)** airports and airways; **(B)** rail stations and railways; and **(C)** roads connecting countries/regions.

Supplementary Figure S2 Learning curves showing the results of training and test loss per epoch for **(A)** the proposed graph convolutional network model with the airway adjacency matrix and **(B)** the multilayer perceptron model.

## Abbreviations

MSE: Mean squared error
RMSE: Root mean squared error
MAE: Mean absolute error
RMSPE: Root mean squared percentage error
MAPE: Mean absolute percentage error.
